# Supplementary material for: Fertilizer Effects on the Nitrogen Isotope Composition of Soil and Different Leaf Locations of Potted Camellia sinensis over a Growing Season
Source: Plants (Basel). 2024 Jun 13;13(12):1628. doi: 10.3390/plants13121628 (PMC11207308; doi:10.3390/plants13121628)
Supplement: Supplementary file 1 [file plants-13-01628-s001.zip › plants-2936224-supplementary.pdf]

Supplementary Tables

Table S1. Nitrogen content (%) of top tea leaves treated with different fertilizers.

Treatment		mean %N ± SD							
		2022 Oct.20	2022 Nov.18	2023 Feb.17	2023 Mar.17	2023 Apr.21	2023 May.10	2023 May.20	2023 May.30
**OF**	Chicken manure	2.5±0.4a	3.0±0.4a	2.1±0.1ab	2.1±0.2ab	2.8±0.5b	2.9±0.1b	2.4±0.5b	2.1±0.2c
	Rapeseed cake	2.6±0.2a	3.0±0.5a	2.5±0.4a	2.7±0.8a	3.4±0.6ab	2.9±0.1b	1.9±0.2b	2.2±0.1c
	Cow manure	2.8±0.3a	3.4±0.2a	2.2±0.3ab	2.1±0.2ab	3.1±0.5b	2.6±0.2bc	2.6±0.4ab	2.8±0.6b
**CF**	Urea	3.0±0.4a	3.5±0.3a	1.9±0.1b	1.7±0.1b	2.4±1.1b	2.3±0.2c	2.6±0.4ab	2.4±0.1bc
**Control**	No fertilizer	2.7±0.4a	3.2±0.6a	2.3±0.3ab	2.6±0.5a	4.4±0.3a	3.9±0.4a	3.2±0.1a	3.3±0.1a

Note: lowercase letters "a, b, c" indicate significant differences between fertilizers (p < 0.05).

Table S2. Nitrogen content (%) of soils treated with different fertilizers.

Treatment		mean %N ± SD							
		2022 Oct.20	2022 Nov.18	2023 Feb.17	2023 Mar.17	2023 Apr.21	2023 May.10	2023 May.20	2023 May.30
**OF**	Chicken manure	1.7±0.2a	1.9±0.2ab	1.8±0.1a	2.0±0.2a	1.8±0.1ab	2.1±0.1a	2.1±0.2ab	1.7±0.2a
	Rapeseed cake	2.0±0.1a	2.2±0.3a	1.8±0.2a	1.9±0.2ab	2.3±0.3a	2.3±0.3a	2.1±0.2a	1.8±0.0a
	Cow manure	1.4±0.4ab	1.4±0.1b	1.2±0.1a	1.4±0.2bc	1.5±0.3bc	1.4±0.1b	1.5±0.3ab	1.4±0.2ab
**CF**	Urea	0.8±0.1b	1.4±0.1b	1.2±0.2a	1.3±0.1c	1.2±0.1c	1.2±0.1b	1.3±0.1b	1.0±0.1b
**Control**	No fertilizer	1.3±0.6ab	1.4±0.6b	1.2±0.8a	1.1±0.5c	1.3±0.5bc	1.3±0.6b	1.4±0.8ab	1.0±0.5b

Note: lowercase letters "a, b, c" indicate significant differences between fertilizers (p < 0.05).
